# Re-visiting the relationship between neighbourhood environment and BMI: an instrumental variables approach to correcting for residential selection bias

**DOI:** 10.1186/1479-5868-10-27

**Published:** 2013-02-20

**Authors:** Cathleen D Zick, Heidi Hanson, Jessie X Fan, Ken R Smith, Lori Kowaleski-Jones, Barbara B Brown, Ikuho Yamada

**Affiliations:** 1 Department of Family and Consumer Studies, University of Utah, 225 South 1400 East, Room 220, 84112, Salt Lake City, UT, USA; 2 Department of Sociology, University of Utah, 225 South 1400 East, Room 220, Utah 84112, Salt Lake City, USA; 3 Population Science, Huntsman Cancer Institute, 2000 Circle of Hope, 84112, Salt Lake City, UT, USA; 4 Interfaculty Initiative in Information Studies, University of Tokyo, 7-3-1 HONGO, 113-0033, Bunkyo-ku, Tokyo, Japan

**Keywords:** Body mass index, Obesity risk, Neighbourhood walkability, Residential selection

## Abstract

**Background:**

A burgeoning literature links attributes of neighbourhoods’ built environments to residents’ physical activity, food and transportation choices, weight, and/or obesity risk. In cross-sectional studies, non-random residential selection impedes researchers’ ability to conclude that neighbourhood environments cause these outcomes.

**Methods:**

Cross-sectional data for the current study are based on 14,689 non-Hispanic white women living in Salt Lake County, Utah, USA. Instrumental variables techniques are used to adjust for the possibility that neighbourhoods may affect weight but heavier or lighter women may also choose to live in certain neighbourhoods. All analyses control for the average BMI of siblings and thus familial predisposition for overweight/obesity, which is often an omitted variable in past studies.

**Results:**

We find that cross-sectional analyses relating neighbourhood characteristics to BMI understate the strength of the relationship if they do not make statistical adjustments for the decision to live in a walkable neighbourhood. Standard cross-sectional estimation reveals no significant relationship between neighbourhood walkability and BMI. However, the instrumental variables estimates reveal statistically significant effects.

**Conclusions:**

We find evidence that residential selection leads to an understatement of the causal effects of neighbourhood walkability features on BMI. Although caution should be used in generalizing from research done with one demographic group in a single locale, our findings support the contention that public policies designed to alter neighbourhood walkability may moderately affect the BMI of large numbers of individuals.

## Background

A burgeoning literature links attributes of neighbourhoods’ built environments to residents’ physical activity, food choices, weight, and/or obesity risk. While these studies do not necessarily view the relationship as causal, it is sometimes implied. If the neighbourhood built environment influences residents’ physical activity, food choices, and/or weight, then changing the built environment may be an important public policy tool that could help reduce Americans’ rising overweight and obesity risk. But what if people choose to live in neighbourhoods that support their dietary and physical activity preferences? This latter view has recently been espoused by land-use developers [1]. Different public policy implications would arise depending upon which mechanism is correct. Do environments affect weight or are weight and residential selection simultaneously determined?

Cross-sectional studies are especially disadvantaged in their ability to draw conclusions about causal relationships between neighbourhood environments and overweight/obesity risk. Analyses of non-experimental data gathered at a single point in time have the potential to contain residential self-selection biases [2]. As such, they may misstate the underlying causal relationship between neighbourhood environments and health-related outcomes such as physical activity, transportation mode choices, dietary intake, and/or healthy body weight. Although authors typically note this cross-sectional limitation [3-5], rarely do they invoke any of the statistical techniques designed to adjust for such self-selection.

Cross-sectional estimates of the association between neighbourhood walkability (measured by a range of variables) and BMI are typically small and statistically significant. For instance, in an analysis of adolescents’ BMI, Ewing and his colleagues find that a one unit increase in a county level sprawl index (i.e., a change toward more compact development) is significantly associated with a .003 decline in an adolescent’s risk of being overweight, holding other factors constant [6]. These very modest sprawl effect sizes are also found in studies of adults [7,8]. While the estimated effects of neighbourhood characteristics on BMI are small, the relationships are nonetheless important from the perspective of policymakers as changes in neighbourhood characteristics have the potential to affect the weight of thousands of residents.

Researchers typically acknowledge that residential selection may confound estimates of the causal relationship between the built environment and behaviours associated with healthy body weight. When available, they exploit the time ordering of longitudinal data to generate improved estimates of causal effects. The results of these longitudinal studies are mixed. Some studies find little or no evidence of a causal relationship between the built environment and physical activity or healthy body weight [9-11] while others find evidence of a reciprocal causal relationship, supporting both environmental and selection influences [7,8,12-16]. Investigations that compare cross-sectional analyses with longitudinal assessments find that statistical relationships between the built environment and physical activity or healthy body weight, sometimes change from significant to insignificant or vice versa when moving from cross-sectional to longitudinal analyses [6,10,17,18]. This mixed evidence may reflect the small, sometimes idiosyncratic samples that often form the basis of these investigations. Regardless of the reasons, the existing longitudinal studies provide no clear consensus on the causal relationship between the built environment and physical activity, transportation mode choice, and/or healthy body weight.

Perhaps not surprisingly, there are a large number of cross-sectional studies that investigate the built environment and various outcomes related to healthy weight. According to recent reviews, few of these studies adopt procedures to assess the effects of residential selection [19,20]. The few cross-sectional studies that do make adjustments adopt one of two general strategies. The first strategy is to use information about residential preferences to disentangle the cross-sectional relationships. Most often this information is acquired through survey questions (e.g., asking about the importance of having stores within walking distance of one’s home). Variables measuring these preferences are included as controls in the empirical work that relates features of the built environment to the outcomes of interest [18,21-27]. Infrequently, researchers have attempted to adjust for residential preferences by controlling for unobserved heterogeneity [28,29] or by comparing the estimates for individuals who can act on their residential preferences (e.g., young adults) to individuals who are far less able to act on their preferences (e.g., adolescents) [30].

Most of the studies that make use of preference information are focused on questions regarding how the built environment affects transportation choices [24,25,29,31] and consequently they are only marginally relevant to our outcome of interest. More germane to the question at hand are studies where the outcome is physical activity and/or some measure of healthy body weight. These studies report that the relationship between the built environment and physical activity/BMI declines in magnitude and statistical significance once one adjusts for preferences [14,23,28,30].

Results of studies that rely on direct questions represent some progress in addressing the residential selection issue. But, as Mokhtarian and Cao [32] highlight, such surveys may be trading off smaller sample size for greater detail on residential preferences. In addition, direct questions used to map residential preferences may generate new sources of bias if respondents’ answers are prone to error because post-relocation preferences are distorted by memory, dissonance reduction, and/or social desirability, or if preferences are endogenous with residential selection.

Often researchers working with cross-sectional data do not have measures of residential preferences or they may conclude that the measures they do have are subject to the measurement biases described above. In those instances, analysts turn to statistical strategies to control for residential selection bias. Several statistical methods have been proposed to make this adjustment in the cross-section including propensity scores and structural equation modelling [12,29,32]. Propensity scores create equivalent groups of “treatment” and “control” individuals by matching groups on multiple sources of differences under the assumption that there is no correlation between the unobservable characteristics and the outcome of interest (i.e., BMI). Structural equations modelling, often utilizing a two-stage least squares or a full-information maximum likelihood approach, corrects for selection by the use of variables called instruments. By definition, the instruments must be variables that relate to proposed predictors, such as choice of neighbourhood, but not to outcomes, such as BMI. Both approaches have advantages and disadvantages [32,33] and the approach selected is often based on data availability.

In the current study, we build on the existing literature that makes use of cross-sectional data to assess the causal effect of neighbourhood characteristics on BMI by incorporating corrections for residential selection using an instrumental variables modelling approach. Specifically, we make use of two-stage least squares techniques to adjust for the possible endogeneity of neighbourhood selection and BMI. We ask whether and to what extent controlling for the effect of residential selection alters our estimates of neighbourhood walkability effects on BMI and overweight/obesity risk. We discuss the implications of our findings for researchers and policymakers concerned about reducing Americans’ overweight/obesity risk.

## Methods

### The model

Our empirical work is informed by household production theory [34-36]. Proponents of this theoretical framework argue that the choice to live in a walkable neighbourhood is likely a function of a range of factors including life cycle stage, housing amenities, housing costs, proximity to institutions such as schools, work, and churches, and preferences regarding physical activity. Likewise, an individual’s weight is hypothesized to be influenced by myriad factors including heredity, norms regarding diet and exercise developed in childhood, food availability within a neighbourhood, and the opportunities to be physically active. In this context, neighbourhood features influence the choices people make about what they eat and/or how physically active they are, which in turn affect BMI. The key insight gained from this model is that choices about residing in a neighbourhood with many or few amenities that support physical activity and choices about weight status are hypothesized to be simultaneously determined. In the absence of correcting for such simultaneity, theory suggests that the parameter estimates of empirical models will be biased because of residential self-selection.

Mathematically, most cross-sectional models assessing the effects of neighbourhood characteristics on BMI implicitly use the following general form to test hypotheses:

(1) $$\mathrm{BMI}=b\left(R,\underline{D}\right)+e$$

where BMI is the body mass index, R is a summary measure of neighbourhood walkability characteristics hypothesized to affect BMI, **D** is a vector of bio-demographic variables that are hypothesized to affect individual BMI and residential selection (e.g., genetic and cultural factors, and e is the error term.

If residential selection forces not captured in **D** exist, then the ordinary least squares (OLS) parameter estimates associated with R in equation (1) will be biased and inconsistent [37]. This statistical problem arises because R is not independent of e. In the case of residential selection, if individuals elect to live in neighbourhoods that support their preferences for physical (in)activity, then the estimated coefficients associated with R in equation (1) will be biased upward. Alternatively, if individuals elect to live in neighbourhoods that do not support their preferences for physical (in)activity, then the estimated coefficients associated with R in equation (1) will be biased downward. In the former situation there will be a tendency to overstate the impact of residential features on BMI by attributing the selection effect to residential features while in the latter case there would be an understatement of the impact of residential features on BMI.

Household production theory suggests that in the presence of residential selection, the appropriate structural model is:

(2) $$R=r\left(\underline{D},\underline{Z}\right)+e_{1}$$

(3) $$\mathrm{BMI}=b\left(\underline{D},R\right)+e_{2}$$

where

**Z** is a vector of variables capturing neighbourhood characteristics that influence the decision to live in a walkable neighbourhood but not BMI (e.g., neighbourhood amenities such as churches, schools, demographic make-up of the neighborhood), e_1_ is the error term for the residential neighbourhood walkability equation, e_2_ is the error term for the BMI equation, and e_1_ and e_2_ are correlated.

All other variables are defined as before. If **Z** contains more than one variable, then the structural model denoted by equations (2) and (3) is over-identified and two-stage least squares or full-information maximum likelihood becomes the optimal estimation strategy.

We elect to use the two-stage least squares estimation in the empirical analyses that follow because we can adjust for the clustering of observations within neighbourhoods using this approach (i.e., by estimating Huber-White standard errors) but not using the full-information maximum likelihood approach^a^. The two-stage least squares approach involves estimating the parameters of the reduced form residential location equation first. From these estimates, a predicted value for neighbourhood walkability, $\hat{R}$, is generated. $\hat{R}$ replaces R in the list of regressors for structural equation (3) which can then be estimated yielding unbiased coefficients [37].

### Data: the Utah population database (UPDB)

The Utah Population Database (UPDB) is one of the world’s richest sources of linked population-based information used in demographic, genetic, epidemiological, and public health research. It forms the core data source for our empirical work as one of its elements is a complete set of Utah birth certificates from 1942–2008. The birth certificates contain health and socio-demographic information for the mother, the father, and the child. Importantly for the purposes of our analyses, these data contain clinical *pre-pregnancy* measures of the mother’s height and weight used to construct her BMI for a large defined population. In addition, the birth certificates provide residential address information allowing us to locate a woman in a specific neighbourhood at the time of the child’s birth. Finally, UPDB and the birth certificates provide information on key measures of **D** including the mother’s age, education, race/ethnicity, marital status, and siblings’ BMI.

The inclusion of a variable measuring siblings’ BMI captures both genetic predispositions for overweight/obesity and the influence of eating and exercise habits acquired in one’s family of origin. This potentially important component of **D** has been absent from all past studies of neighbourhood characteristics and BMI but has been identified as an important covariate in one other study that examined the relationship between social networks and BMI [38].

The information regarding familial relationships in UPDB allows us to link individuals in the current study to both their female and male siblings who have a Utah driver license. The driver license record contains information on self-reported height and weight along with age, gender, and year that the license was issued. For those subjects who have one or more siblings in UPDB, we use this information to construct the average standardized sibling residual BMI (SIBBMI). This variable measures the average number of standard deviations away from the age-year-specific predicted value for siblings with a driver license.

Our sample includes women age 21 or older living in Salt Lake County with a pre-pregnancy BMI between 18.5 and 49.9 who have had a first birth during 1995–2005. We omit young mothers (< age 20) because they are more likely to be unmarried and living in their family of origin making residential selection a non-issue for them [30]. The sample comprises first birth mothers because discussions about starting a family may lead parents to re-consider their residential choice. The choices parents make because of child-based factors may be a driving force in affecting location decisions that in turn affect maternal BMI. In addition, by limiting the sample to first birth mothers we avoid attributing post-pregnancy weight gain that occurs for many women [39] to their residential choice. Underweight women (i.e., BMI < 18.5, N = 3,784) and extremely obese women (i.e., BMI > 49.9, N = 48) are excluded because they may have complicating health conditions that are associated with their extreme weights. The sample includes only white, non-Hispanic women in order to hold race/ethnicity factors constant and we exclude 407 women who are missing geocoded residential information. This study examines the 1995–2005 period because we link the information from the birth certificates to data on neighbourhood characteristics from the 2000 Census for Salt Lake County. Both the full sample (N = 35,685) and a sample restricted to those women who have one or more siblings with driver license data were examined in order to construct sibling BMI (N = 14,689). The substantive results regarding our tests for selection bias are very similar across the two samples. We elect to present the results that control for sibling BMI since it corrects for potential genetic and family-of-origin effects and it is a variable that has been omitted in previous studies that examine the relationship between neighbourhood characteristics and BMI^b^.

From the birth certificate data, clinically measured height and weight information are converted to BMI ([weight in kg]/[height in m]^2^) as well as a categorical measure of overweight/obese (25.0 ≤ BMI ≤49.9) in relation to healthy weight (18.5 ≤ BMI < 25.0). We also use data from the birth certificates to operationalize the elements of **D**. Specifically, we have information about the woman’s age (AGE), education (EDUC), marital status (MARRIED), and year of her pre-pregnancy weight measurement (BMIYR).

### Linked neighbourhood data

The 2000 U.S. Census contains numerous variables that capture neighbourhood characteristics measured at the Census block group level. The Census block group is a relatively small area (i.e., typically about 1,500 residents, ranging from 300 to 3000) [40] that approximates a local neighbourhood. We use 550 of the 567 census block groups in Salt Lake County, Utah, eliminating 17 block groups because they are at the periphery of the county (e.g., including mountainous areas) and have very few residents who meet our sample requirements.

Key to our analysis is the identification of a summary measure of residential walkability selection, R. We construct a factor score of neighbourhood walkability based on measures of land use diversity, population density, and neighbourhood design features, the so-called 3-D’s identified in past research (see [20] for a review). Specifically, the 3-D block group measures used to construct the factor scores are presented in Table 1. We follow past research in defining the central business district (CBD) and measure each individual’s proximity as the network distance between the centroid of each block group to the closest street intersection in the CBD measured in miles. The remaining variables in Table 1 are based on the 2000 U.S. Census.

**Table 1:** Factor pattern for neighbourhood walkability variables

**Dimensions of neighbourhood walkability measured at the block group level**	**Grand mean (N = 550)**	**Mean for block groups in lowest factor score quartile (N = 138)**	**Mean for block groups in highest factor score quartile (N = 138)**	**Factor loadings**
Proximity to Central Business District (miles)	14.17	23.00	4.66	0.30
Population Density (residents/sq mile)	5,639.51	3,702.62	7,472.63	0.17
Proportion Who Walk to Work	0.02	0.01	0.06	0.24
Proportion Who Bike to Work	0.01	0.00	0.02	0.21
Proportion Who Take Public Transit to Work	0.04	0.02	0.08	0.24
Median Age of Housing (yrs)	29.08	12.79	46.12	0.26

The factor scores presented in Table 1 were derived from a confirmatory factor analysis where only one factor was theorized. The analysis resulted in a standardized Cronbach’s alpha of 0.78, which is above the minimum acceptable level of reliability [41]. The factor scores distinguish high walkable neighbourhoods from low walkable neighbourhoods as shown in the columns that contrast the means for the highest quartiles from the means for the lowest quartiles. In comparison with neighbourhoods that have high factor scores, neighbourhoods with low factor scores are farther away from the central business district, have lower population density, have a smaller proportion of residents who use public transit or active modes of transportation to commute to and from work, and have a younger housing stock.

The **Z** variables are expected to influence the choice of living in a walkable neighbourhood but not BMI. The census block group measures of residential features that fall in this category include number of churches (NCHURCH), number of schools (NSCHOOL), and the proportion of the neighbourhood population under age 16 (UNDER16). Data for the number of churches come from the Utah’s State Geographic Information Database (SGID) [42]. Counts of the number of schools within a block group are drawn from the Utah State Office of Education [43]. The proportion of the population in the census block group who are under age 16 comes from the 2000 Census [40].

To protect confidentiality of individuals in the UPDB, the UPDB staff linked all UPDB data to the census block group information using Universal Transverse Mercator (UTM) coordinates. They then provided the researchers with a data set without names or individual addresses. Use of the data for this project has been approved by the University’s Resource for Genetic and Epidemiologic Research Committee and the University’s Institutional Review Board.

### The analyses

Our data allow us to operationalize and test the following alternative structural models of neighbourhood walkability and BMI. In the first model, tested in equation 4 below, there is no allowance for residential selection effects. The neighbourhood’s walkability factor score is hypothesized to affect BMI as neighbourhoods with higher factor scores are hypothesized to have a constellation of features that promote greater physical activity (e.g., a diversity of destinations as approximated by the proportion of residents who walk to work). In addition to the neighbourhood walkability factor score, we hypothesize that a woman’s BMI is influenced by bio-demographic factors including age, education, marital status, calendar year (reflecting the secular upward trend in BMI), and average familial BMI as measured by the average standardized residual BMIs of a woman’s siblings. We include both age and age-squared to allow for nonlinearity in age effects. Thus, the estimated structural equation for the first model is:

(4) $$\begin{array}{cc} \;\mathrm{BMI}=b & \left(\mathrm{AGE},\mathrm{AGESQ},\mathrm{EDUC},\mathrm{MARRIED},\mathrm{BMIYR},\right)\\ & \;\left(\mathrm{SIBBMI},\mathrm{RESWALK}\right) \end{array}$$

The second model allows for choice of a walkable neighbourhood residence and BMI to be simultaneously determined. With this modification, the two-stage least squares estimation model becomes:

(5) $$\begin{array}{cc} \;\mathrm{RESWALK}=r & \left(\mathrm{AGE},\mathrm{AGESQ},\mathrm{EDUC},\mathrm{MARRIED},\mathrm{BMIYR},\right)\\ & \;\left(\mathrm{SIBBMI},\mathrm{NSCHOOL},\mathrm{NCHURCH},\mathrm{UNDER}16\right) \end{array}$$

(6) $$\begin{array}{cc} \;\mathrm{BMI}=b & \left(\mathrm{AGE},\mathrm{AGESQ},\mathrm{EDUC},\mathrm{MARRIED},\mathrm{BMIYR},\right)\\ & \;\left(\mathrm{SIBBMI},\mathrm{RESWALK}\;\mathrm{PREDICTED}\right) \end{array}$$

Estimation of the above system of equations is done in two stages. In stage one, equation (5) is estimated as a function of all of the exogenous regressors in the system. The predicted values from this first stage estimation are then included in place of the actual walkability factor scores in the second stage regression. We test for endogeneity, the strength of our instruments, and the independence of the instruments from BMI – all of which help us to assess if the instrumental variables approach should be preferred. The models are then re-estimated with the qualitative dependent variable that measures whether the woman is overweight/obese. Analyses are done using STATA 11.0 IVREGRESS and REGRESS procedures with adjustments made for Census block group sample clustering.

## Results

Descriptive information for all of the variables we use in the analyses is shown in Table 2. Women’s average BMI prior to the first pregnancy is slightly more than 24 and almost one-third of the women are overweight or obese. The typical woman is married, age 25.5 and has approximately 14 years of schooling. Most neighbourhoods in which these women live have one school and one church. Almost a quarter of the neighbourhood residents are under age 16.

**Table 2:** Descriptive statistics (N = 14,689)

**Variable**	**Definition**	**Mean/proportion**	**Standard deviation**
Residential walkability and weight measures			
RESWALK	Factor score for neighbourhood walkability	−0.24	0.98
BMI	Weight in kg divided by height in meters squared	24.27	4.88
BMIBG ≥ 25	Proportion of the women with a BMI > 25.0	0.32	0.47
**D** Variables (Exogenous): Hypothesized to affect BMI and residential walkability			
AGE	Age in years	25.58	3.98
EDUC	Years of schooling	13.93	1.88
MARRIED	1 = married	0.89	
BMIYR	Year of pregnancy (1 = 1995… 11 = 2005)	6.02	3.12
SIBBMI	Average age and year standardized sibling BMI residual	−0.03	0.71
**Z** Variables hypothesized to affect residential walkability only			
NSCHOOL	Number of schools	0.61	0.90
NCHURCH	Number of churches	0.91	0.87
LIBRARY	Presence of one or more public libraries (1 = yes)	0.04	0.21
UNDER16	Proportion of the population ≤ Age 15	0.24	0.08

Our first step in the multivariate analyses is to test for the endogeneity of RESWALK and BMI. This involves estimating the reduced form equation where RESWALK is the dependent variable. The residuals from this equation are then included as an additional regressor in the structural equation estimating BMI [44]. The resulting Durbin-Wu-Hausman F-statistic generated from this second equation is a measure of endogeneity. For the current application, that F-statistic is 22.37 (p < .01), evidence that electing to reside in a walkable neighbourhood and BMI are endogenous which supports our hypothesis.

The next step is to test the strength and independence of those instruments used in the first stage and excluded from the second stage estimation. Given that RESWALK is our only endogenous variable, the strength of the instruments can be assessed by computing the joint significance of **Z** in the first stage regression using an F-Statistic [45]. The resulting F-statistic is 91.87 (p < .01) suggesting that our instruments are strong and justified.

Independence of the instruments is assessed by Hansen’s J statistic which has a *χ*^2^ distribution with degrees of freedom equal to the number of over-identifying restrictions [44]. A statistically significant value suggests that the **Z** instruments used in the first stage are *not* independent of BMI. In our models, Hansen’s J is .08 (p = .96), indicating that the **Z** instruments are not associated with BMI.

Having satisfied the criteria for using the instrumental variables approach, we now turn to comparing the instrumental variables estimates to the estimates of the traditional single equation BMI model. These alternative estimates appear in Table 3. The key variable in our alternative models is RESWALK. The estimated coefficient is very small and statistically insignificant. In contrast, the estimated coefficient in the instrumental variables regression is larger and statistically significant. This suggests that empirical investigations of neighbourhood characteristics and BMI that do not account for residential selection may be significantly understating neighbourhood effects. We observe the same pattern of effects and statistical significance in Table 4 where the outcome is overweight/obesity risk.

**Table 3:** Parameter estimates of the alternative bmi structural model specifications (t statistics in parentheses)

**Independent variables**	**Instrumental variables regression coefficients (t-statistics)**	**Least squares regression coefficients (t-statistics)**
INTERCEPT	27.96	28.21
	(82.41)**	(83.45)**
AGE^a^	0.22	0.22
	(16.57)**	(16.66)**
AGE_SQ^a^	−0.00	−0.01
	(−2.71)**	(−2.89)**
EDUC	−0.31	−0.33
	(−13.04)**	(−13.88)**
MARRIED	0.33	0.38
	(2.62)**	(2.97)**
BMIYR	0.09	0.10
	(7.54)**	(8.11)**
SIBBMI	2.34	2.35
	(35.82)**	(35.75)**
RESWALK	−0.24	−0.00
	(−3.23)**	(−0.08)
Adjusted R^2^	0.16	0.16
F Statistic	268.52**	267.90**

**p < .05.

^a^Age is centered before creating age squared so as to avoid any possible collinearity problems associated with entering both age and age squared as regressors.

**Table 4:** Parameter estimates of the alternative structural model specifications for the risk of being overweight/obese

**Independent variables**	**Instrumental variables odds ratios (95% CI)**	**Logistic regression odds ratios (95% CI)**
AGE^a^	1.09	1.09
	(1.07–1.10)	(1.07–1.10)
AGE_SQ^a^	1.01	1.01
	(1.00–1.00)	(1.00–1.00)
EDUC	0.89	0.89
	(0.87–0.91)	(0.87–0.91)
MARRIED	1.11	1.14
	(0.99-1.25)	(1.01–1.28)
BMIYR	1.04	1.04
	(1.03–1.06)	(1.03–1.06)
SIBBMI	2.37	2.38
	(2.23–2.53)	(2.23–2.53)
RESWALK	0.90	1.00
	(0.83–0.97)	(0.96–1.04)
McFadden R^2^	0.08	0.08

^a^Age is centered before creating age squared so as to avoid any possible collinearity problems associated with entering both age and age squared as regressors.

What variables are associated with the choice to live in a neighbourhood with a higher block-group walkability score? To answer that question, we turn to Table 5 which contains the parameter estimates of the reduced form factor score neighbourhood walkability equation. In this table, we see that more churches are associated with a woman’s decision to live in more walkable neighbourhoods while the number of schools and the proportion of the population under age 16 are inversely related to a woman’s decision to live in more walkable neighbourhoods.

**Table 5:** Parameter estimates of the reduced form neighborhood walkability equation

**Independent variables**	**Coefficient (t statistic)**
INTERCEPT	1.35
	(7.64)**
AGE^a^	0.00
	(0.31)
AGE_SQ^a^	0.00
	(1.36)
EDUC	0.02
	(2.64)**
MARRIED	−0.07
	(−2.98)**
BMIYR	−0.02
	(−5.83)**
SIBBMI	0.02
	(1.82)*
NSCHOOL	−0.16
	(−2.29)**
NCHURCH	0.10
	(2.09)**
UNDER16	−6.76
	(−10.68)**
Adjusted R^2^	0.42
F Statistic	43.79**

*p < .10 **p < .05.

^a^Age is centered before creating age squared so as to avoid any possible collinearity problems associated with entering both age and age squared as regressors.

## Discussion

Our research suggests that cross-sectional analyses relating neighbourhood characteristics to BMI may understate the strength of the relationship if statistical adjustments for the endogeneity of BMI and neighbourhood walkability are neglected. Few prior studies have focused on residential selection effects. But, the majority of studies conclude that the causal effects of neighbourhood walkability are over-stated rather than understated in studies that do not correct for residential selection bias. Indeed, with the exception of one longitudinal study [16], our finding is counter to longitudinal [6-8,10] and cross-sectional [30] studies that assess residential selection effects as they relate to BMI and/or physical activity.

The behavioural mechanism behind our results is likely complex. It is generally assumed individuals with healthy body weights prefer to live in walkable neighbourhoods or prefer to live in neighbourhoods that have characteristics that are highly correlated with walkability. But, to the extent that some walkability features are inversely related to other competing dimensions of neighbourhood choice (e.g., quality of schools, less traffic, lower per square foot housing costs), it is plausible that the selection will operate in reverse. In our estimation, it would appear that number of neighbourhood schools and the proportion of the neighbourhood population under age 16 are inversely related to neighbourhood walkability. If these are competing dimensions of neighbourhoods that first-time mothers have strong preferences for, then these considerations may outweigh any preferences for regulating BMI by living in a walkable neighbourhood. This general point has been made by others [16] and we believe it merits further research.

While our study findings are not definitive, we argue that they should not be discounted as the results are robust to a range of alternative instrumental variable specifications (available from the authors upon request).Furthermore, the current analyses differ from past cross-sectional analyses in several important ways. First, we use a structural equations modelling approach rather than the more commonly implemented preference measures that may not adequately address the endogeneity issue [32].

Second, our analyses are based on a large, but rather select group of individuals from one county and we measure neighbourhood features at the block group level. Our choice of geographic location and neighbourhood scale differs from those used in others studies. In some studies the unit of analysis for neighbourhood features has been the county [6-8,10] or zip code [15], while in other studies it has been a smaller unit within a specific urban/suburban area (e.g., Atlanta, GA, Alameda County, CA) [23,28]. Previous research suggests that the choice of neighbourhood scale may influence the conclusions drawn regarding the relationship between neighbourhood features and BMI [46]. While it is unclear what the optimal geographic scale is for measuring neighbourhood walkability, a census block group in urban areas is more likely to represent walkable distances than a county or zip code. Moreover, as with other place-specific studies, the generalizability of our findings may be limited. Thus, rather than discounting the current findings, we believe our empirical work reinforces the need for the estimation of additional models with other samples that use statistical controls for residential selection.

Absent the ability to implement randomized field experiments (where individuals are randomly assigned to neighbourhoods), researchers will continue to struggle to answer the question of whether neighbourhood features can facilitate healthy body weight. The best strategy is to implement statistical designs that adjust for residential selection using data from a range of communities. These models should be tested with samples of both men and women and, where the data allow, attention should be given to the choice of geographic scale for measuring neighbourhood effects. Such research would help to build a consensus regarding the causal relationship between walkable neighbourhoods and BMI.

The results of the current investigation suggest that the residential location choices of young, white, non-Hispanic women in Salt Lake County are likely complicated. Less walkable neighbourhoods also typically have lower housing costs (measured on a per square foot basis), newer homes, newer schools, and more young families. The attractiveness of these neighbourhood features may outweigh walkability considerations for many women.

Our estimates of the absolute effects of a change in neighbourhood walkability on BMI and overweight/obesity risk are modest. A one unit increase in the factor score (equivalent from moving from a neighbourhood in the 25^th^ percentile for walkability to a neighbourhood in the 74^th^ percentile for walkability) is associated with a .36 decline in BMI. This translates into about a three pound weight difference for a woman who is 5 feet 4 inches tall. While this is a modest effect size, across the 1500–3000 people living in a typical neighbourhood, the total weight difference could be substantial. Moreover, the change in overweight/obesity risk reduction is larger, with a one unit change in the factor score associated with a 10 per cent reduction in the risk of being overweight/obese. In this context, public policies designed to improve the walkability of new neighbourhoods so as to mimic the walkability features of older neighbourhoods (e.g., decisions regarding public transit routes, the inclusion of trees in street-side landscaping, zoning laws regarding mixed land use) have the potential to affect the incremental BMI of many individuals.

Our estimates also reveal the novel, but not surprising finding, that familial effects on BMI are large, *ceteris paribus*. Future research should focus on disentangling the genetic components of this relationship from the environmental components. If the environmental components of the family of origin dominate, then this would have implications for the importance of early intervention. That is, if the exercise and eating habits that siblings learn in their families of origin have effects on BMI throughout adulthood, then interventions directed at improving children’s physical activity and nutrition habits may generate returns across the entire life course.

## Conclusions

We find evidence that residential selection bias understates the relationship between neighbourhood walkability features and BMI. Although caution should be used in generalizing from research done with one group in a single locale, our findings support the contention that public policies designed to alter neighbourhood walkability may moderately affect residents’ BMI. Despite the moderate effect size, such policies are appealing because they have the potential to affect large numbers of individuals.

## Endnotes

^a^We actually estimated both. The coefficients do not differ significantly across the estimation approaches but the standard errors change when we adjust for the clustering.

^b^The results based on the full sample are available from the authors upon request.

## Competing interests

The authors declare they have no competing interests.

## Authors’ contributions

All authors participated in discussions regarding this research project and contributed to the development of the empirical approach and the interpretation of the results. HH and IY constructed the GIS-based measures that were linked to the data files. CDZ estimated the empirical models, wrote the first draft of the manuscript, and the revision. All authors have read and approved the final manuscript.

## References

[B1] Clark MI Berry TR Spence JC Nykiforuk C Carlson M Blanchard C Key stakeholder perspectives on the development of walkable neighbourhoods Health Place 2010 16 43 50 10.1016/j.healthplace.2009.08.001 19733495 PMC5003602

[B2] Oakes JM Commentary: Advancing neighbourhood-effects research - selection, inferential support, and structural confounding Int J Epidemiol 2006 35 643 647 10.1093/ije/dyl054 16556642

[B3] Owen N Humpel N Leslie E Bauman A Sallis JF Understanding environmental influences on walking - Review and research agenda Am J Prev Med 2004 27 67 76 10.1016/j.amepre.2004.03.006 15212778

[B4] Smith KR Brown BB Yamada I Kowaleski-Jones L Zick CD Fan JX Walkability and Body Mass Index: Density, design, and new diversity measures Am J Prev Med 2008 35 237 244 10.1016/j.amepre.2008.05.028 18692736

[B5] Zick CD Smith KR Fan JX Brown BB Yamada I Kowaleski-Jones L Running to the store? The relationship between neighborhood environments and the risk of obesity Soc Sci Med 2009 69 1493 1500 10.1016/j.socscimed.2009.08.032 19766372 PMC2791711

[B6] Ewing R Brownson RC Berrigan D Relationship between urban sprawl and weight of United States youth Am J Prev Med 2006 31 464 474 10.1016/j.amepre.2006.08.020 17169708 PMC1880893

[B7] Plantinga AJ Bernell S The association between urban sprawl and obesity: Is it a two-way street? J Reg Sci 2007 47 857 879 10.1111/j.1467-9787.2007.00533.x

[B8] Plantinga AJ Bernell S Can urban planning reduce obesity? The role of self-selection in explaining the link between weight and urban sprawl Rev Agric Econ 2007 29 557 563 10.1111/j.1467-9353.2007.00370.x

[B9] Berry TR Spence JC Blanchard C Cutumisu N Edwards J Nykiforuk C Changes in BMI over 6 years: the role of demographic and neighborhood characteristics Int J Obes 2010 34 1275 1283 10.1038/ijo.2010.36 PMC5005067 20157324

[B10] Lee IM Ewing R Sesso HD The built environment and physical activity levels: the Harvard alumni health study Am J Prev Med 2009 37 293 298 10.1016/j.amepre.2009.06.007 19765500 PMC2749578

[B11] Eid J Overman HG Puga D Turner MA Fat city: Questioning the relationship between urban sprawl and obesity J Urban Econ 2008 63 385 404 10.1016/j.jue.2007.12.002

[B12] Boone-Heinonen J Gordon-Larsen P Guilkey DK Jacobs DR Jr Popkin BM Environment and physical activity dynamics: The role of residential self-selection Psychol Sport Exerc 2011 12 54 60 10.1016/j.psychsport.2009.09.003 21516236 PMC3079234

[B13] MacDonald JM Stokes RJ Cohen DA Kofner A Ridgeway GK The effect of light rail transit on body mass index and physical activity Am J Prev Med 2010 39 105 112 10.1016/j.amepre.2010.03.016 20621257 PMC2919301

[B14] Handy S Cao X Mokhtarian PL Self-selection in the relationship between the built environment and walking: Empirical evidence from Northern California J Am Plann Assoc 2006 72 55 74 10.1080/01944360608976724

[B15] Gibson DM The neighborhood food environment and adult weight status: estimates from longitudinal data Am J Public Health 2011 101 71 78 10.2105/AJPH.2009.187567 21088263 PMC3000723

[B16] Boone-Heinonen J Guilkey DK Evenson KR Gordon-Larsen P Residential self-selection bias in the estimation of built environment effects on physical activity between adolescence and young adulthood Int J Behav Nutr Phys Activ 2010 7 70 70 10.1186/1479-5868-7-70 PMC2959083 20920341

[B17] Handy S Cao XY Mokhtarian P Correlation or causality between the built environment and travel behavior? Evidence from Northern California Transp Res Part D: Transp Environ 2005 10 427 444 10.1016/j.trd.2005.05.002

[B18] Berry TR Spence JC Blanchard CM Cutumisu N Edwards J Selfridge G A longitudinal and cross-sectional examination of the relationship between reasons for choosing a neighbourhood, physical activity and body mass index Int J Behav Nutr Phys Activ 2010 7 57 57 10.1186/1479-5868-7-57 PMC2910030 20602776

[B19] Papas MA Alberg AJ Ewing R Helzlsouer KJ Gary TL Klassen AC The built environment and obesity Epidemiol Rev 2007 29 129 143 10.1093/epirev/mxm009 17533172

[B20] Feng J Glass TA Curriero FC Stewart WF Schwartz BS The built environment and obesity: A systematic review of the epidemiologic evidence Health Place 2010 16 175 190 10.1016/j.healthplace.2009.09.008 19880341

[B21] Cao XY Handy SL Mokhtarian PL The influences of the built environment and residential self-selection on pedestrian behavior: Evidence from Austin, TX Transportation 2006 33 1 20 10.1007/s11116-005-7027-2

[B22] Cao XY Mokhtarian PL Handy SL Cross-sectional and quasi-panel explorations of the connection between the built environment and auto ownership Environ Plan A 2007 39 830 847 10.1068/a37437

[B23] Frank LD Saelens BE Powell KE Chapman JE Stepping towards causation: Do built environments or neighborhood and travel preferences explain physical activity, driving, and obesity? Soc Sci Med 2007 65 1898 1914 10.1016/j.socscimed.2007.05.053 17644231

[B24] Scheiner J Social inequalities in travel behaviour: trip distances in the context of residential self-selection and lifestyles J Transp Geogr 2010 18 679 690 10.1016/j.jtrangeo.2009.09.002

[B25] Bagley MN Mokhtarian PL The impact of residential neighborhood type on travel behavior: A structural equations modeling approach Ann Reg Sci 2002 36 279 297 10.1007/s001680200083

[B26] Schwanen T Mokhtarian PL What if you live in the wrong neighborhood? The impact of residential neighborhood type dissonance on distance traveled Transp Res Part D: Transp Environ 2005 10 127 151 10.1016/j.trd.2004.11.002

[B27] Sallis JF Saelens BE Frank LD Conway TL Slymen DJ Cain KL Chapman JE Kerr J Neighborhood built environment and income: Examining multiple health outcomes Soc Sci Med 2009 68 1285 1293 10.1016/j.socscimed.2009.01.017 19232809 PMC3500640

[B28] Pinjari AR Bhat CR Hensher DA Residential self-selection effects in an activity time-use behavior model Trans Res Part B: Met 2009 43 729 748 10.1016/j.trb.2009.02.002

[B29] Bhat CR Guo JY A comprehensive analysis of built environment characteristics on household residential choice and auto ownership levels Trans Res Part B: Met 2007 41 506 526 10.1016/j.trb.2005.12.005

[B30] Smith KR Zick CD Kowaleski-Jones L Brown BB Yamada I Fan JX Effects of neighborhood SES and walkability on obesity: Comparing adolescents and young adults to assess selection and causal influences Soc Sci Res 2012 40 1445 1455 21841846 10.1016/j.ssresearch.2011.04.009 PMC3153141

[B31] Schwanen T Mokhtarian P What affects commute mode choice: neighborhood physical structure or preferences toward neighborhoods? J Transp Geogr 2005 13 83 99 10.1016/j.jtrangeo.2004.11.001

[B32] Mokhtarian PL Cao X Examining the impacts of residential self-selection on travel behavior: A focus on methodologies Trans Res Part B: Met 2008 42 204 228 10.1016/j.trb.2007.07.006

[B33] Gibson-Davis C Foster EM A cautionary tale: Using propensity scores to estimate the effect of food stamps on food insecurity Soc Sci Rev 2006 80 93 126

[B34] Becker GS A theory of the allocation of time Econ J 1965 75 493 517 10.2307/2228949

[B35] Becker GS A treatise on the family 1991 enlarged Cambridge: Harvard University Press

[B36] Cawley J An economic framework for understanding physical activity and eating behaviors Am J Prev Med 2004 27 117 125 10.1016/j.amepre.2004.06.012 15450622

[B37] Green WH Econometric Analysis 1993 2 New York: Macmillan Publishing Company

[B38] Christakis NA Fowler JH The spread of obesity in a large social network over 32 years New England J Med 2007 357 370 379 10.1056/NEJMsa066082 17652652

[B39] Rooney BL Schauberger CW Excess Pregnancy Weight Gain and Long Term Obesity: One Decade Later Obstet Gynecol 2002 100 245 252 10.1016/S0029-7844(02)02125-7 12151145

[B40] U.S. Census Bureau Demographic Profile Highlights, Summary File 3 2000 2000 Salt Lake County, Utah: Census http://factfinder.census.gov

[B41] Nunnally JC Psychometric theory 1978 2 New York: McGraw-Hill

[B42] Utah AGRC Utah's State Geographic Information Database 2011 Salt Lake City: State of Utah

[B43] Utah State Office of Education 2010 Salt Lake City

[B44] Baum C Schaffer M Stillman S Instrumental variables and GMM: Estimation and testing Stata J 2003 3 1 31

[B45] Bound J Jaeger DA Baker RM Problems with Instrumental Variables Estimation When the Correlation between the Instruments and the Endogenous Explanatory Variable Is Weak J Am Stat Assoc 1995 90 443 450

[B46] Timperio A Jeffery RW Crawford D Roberts R Giles-Corti B Ball K Neighbourhood physical activity environments and adiposity in children and mothers: a three-year longitudinal study Int J Behav Nut Phys Activ 2010 7 18 18 10.1186/1479-5868-7-18 PMC2847539 20170507

